# A Yeast Model for Understanding ALS: Fast, Cheap, and Easy to Control

**DOI:** 10.1371/journal.pbio.1001053

**Published:** 2011-04-26

**Authors:** Richard Robinson

**Affiliations:** Freelance Science Writer, Sherborn, Massachusetts, United States of America

**Figure pbio-1001053-g001:**
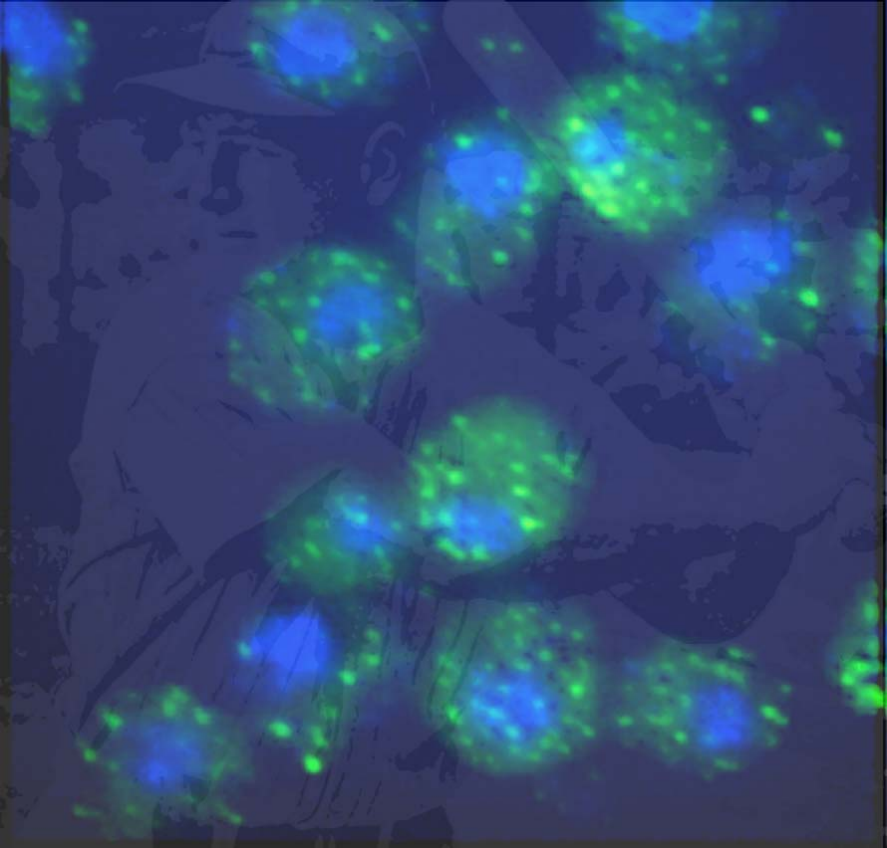
Superimposed on a picture of Lou Gehrig at bat is an image of a yeast model of proteotoxicity in the disease that bears his name. Human FUS/TLS, normally found in the nucleus (blue), is mislocalized as punctate cytoplasmic inclusions (green), just as in some cases of Lou Gehrig's Disease.

[Fig pbio-1001053-g001]Amyotrophic lateral sclerosis (ALS) is a neurodegenerative disease in which the motor neurons of the central nervous system—those cells of the brain and spinal cord that control muscles—die off. The resulting paralysis typically leads to death within 3–5 years of onset. The cause of the disease in the majority of cases is completely unknown, and there is no treatment that halts or significantly slows the disease. In the United States ALS is often known as Lou Gehrig's Disease, after the baseball player who famously suffered from it.

Several genes have been linked to ALS. The most recent, called FUS (fused in sarcoma, a reference to the context of its discovery), is the subject of two new studies in this issue of *PLoS Biology*, one by Zhihui Sun, Zamia Diaz, James Shorter, Aaron Gitler, and colleagues, and the other by Shulin Ju, Gregory Petsko, Dagmar Ringe, and colleagues. Both explore FUS biology in yeast, and highlight the potential for modeling elements of complex diseases in this simplest of eukaryotic cell systems. Results from both studies suggest that defects in RNA processing and transport may be a central element of ALS pathophysiology.

Within the cytoplasm of motor neurons in ALS patients, proteins aggregate to form insoluble clumps, called inclusions, which can include both FUS and another ALS-causing protein, called TDP-43. When the two research groups overexpressed human FUS in yeast, they observed cytoplasmic inclusions. Inclusions form in most of the neurodegenerative diseases, including Alzheimer's disease and Parkinson's disease, suggesting that common defects in protein handling may link all of them.

In humans, FUS is found predominantly in the nucleus, and at least some ALS-associated mutations reduce the nuclear/cytoplasmic ratio of the protein, suggesting that its mislocalization to the cytoplasm, rather than mutation per se, may be an important step in disease pathogenesis. Supporting that hypothesis, both groups found that restricting overexpressed wild-type FUS to the nucleus mitigated its toxic effect.

Both FUS and TDP-43 are RNA-binding proteins. But Sun et al. found that purified FUS is far more prone to aggregation than purified TDP-43, and both groups showed that the molecular features that are critical for aggregation differed between the two proteins, which may indicate that the disease-causing mechanism also differs between them, despite their broadly similar functions.

Both groups conducted genome-wide screens to identify genes that specifically mitigate toxicity. Gratifyingly, despite differences in lab and protocol details, there was a large overlap between the two sets of candidates, suggesting these genes play central roles in bypassing whatever the toxic pathways are. The handful of identified genes included ones coding for other DNA/RNA binding proteins. One, called ECM32, has a human homolog, hUPF1, that Ju et al. found also rescued toxicity. One function of hUPF1 is in messenger RNA quality control, strengthening the case that RNA handling is defective in FUS-caused ALS. Interestingly, expression of hUPF1 was able to rescue FUS toxicity in yeast without driving FUS out of the inclusions or sending it back to the nucleus, suggesting that it may be possible to overcome the effects of mislocalized FUS therapeutically without solving the difficult problem of restoring it to its proper compartment.

These two studies have at least two important consequences. By identifying new genes that can lessen ALS-linked toxicity, they point the way to exploration of new therapeutics based on RNA processing. Perhaps just as importantly, they demonstrate that yeast has the potential to be a versatile system for modeling aspects of ALS that previously have only been modeled in mice. Since testing ideas about pathogenesis and treatment is much faster and cheaper in yeast, these results may open the way for more rapid progress in understanding the disease, its treatment, and the role of this new gene in ALS development.

**Sun Z, Diaz Z, Fang X, Hart MP, Chesi A, et al. (2011) Molecular Determinants and Genetic Modifiers of Aggregation and Toxicity for the ALS Disease Protein FUS/TLS. doi: 10.1371/journal.pbio.1000614**

**Ju S, Tardiff DF, Han H, Divya K, Zhong Q, et al. (2011) A Yeast Model of FUS/TLS-Dependent Cytotoxicity. doi:10.1371/journal.pbio.1001052**

